# Combined endoscopic and long ileus tube treatment for giant gastric bezoars with ileus in a child

**DOI:** 10.1055/a-2879-0348

**Published:** 2026-05-28

**Authors:** Le Zhang, Xiao-Li Fu, Meng Shi, Xu-Xia Wei

**Affiliations:** 1 Gastroenterology 576219 Children's Hospital Affiliated to Shandong UniversityJinanShandongChina

While endoscopic fragmentation is established for gastric bezoars, its combination with a long ileus tube for concurrent small bowel obstruction, especially in pediatric cases, is rarely documented. This case demonstrates the efficacy of this combined minimally invasive approach.

**Fig. 1:**
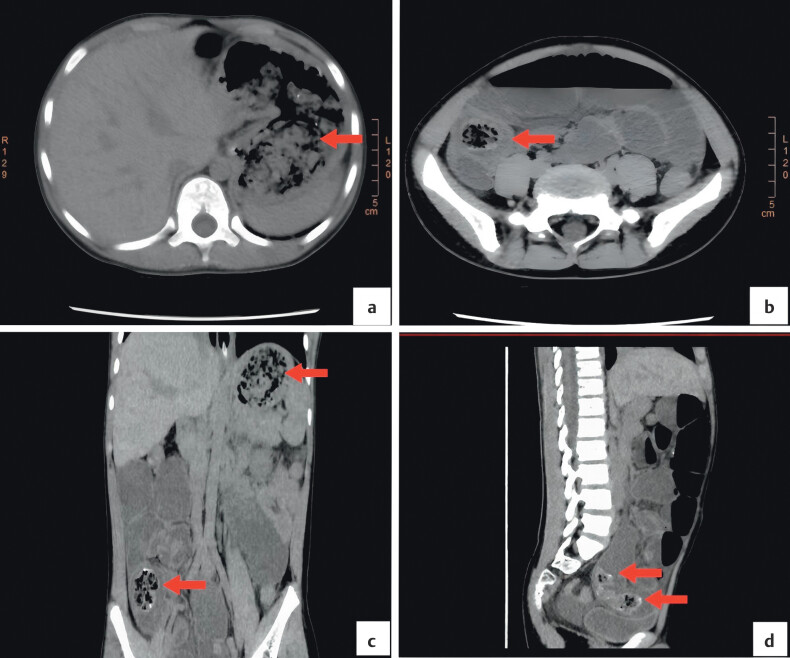
Abdominal CT is showing a huge gastric bezoar (5.7×4.5×6.0 cm) in the stomach, and small bowel ileus with multiple gastric bezoars (The maximum is 3.7×2.5×2.6 cm.). CT, computed tomography.

**Fig. 2:**
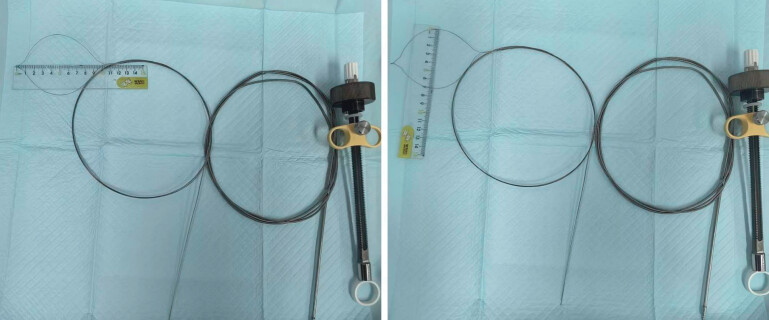
A gastric bezoar lithotripsy cutter with a 70×100 mm snare.

**Fig. 3:**
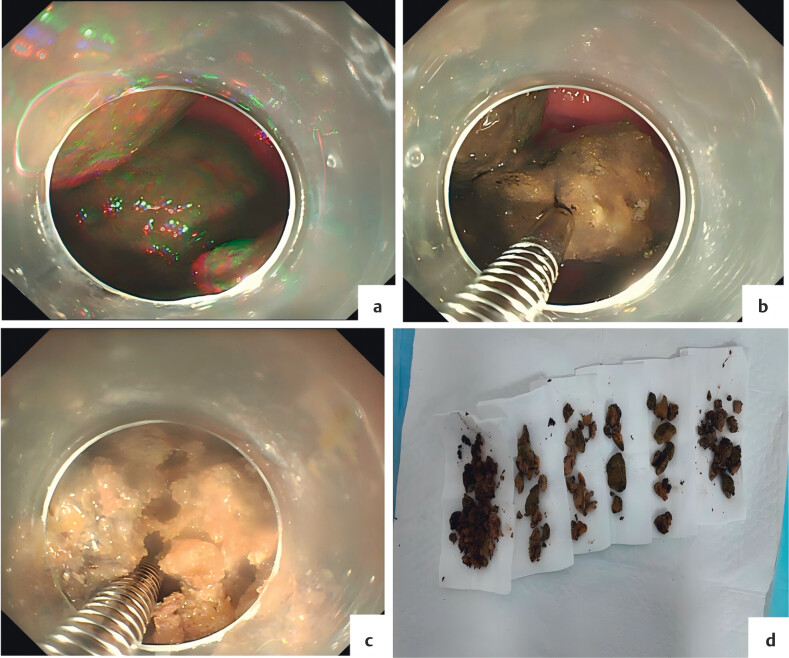
( **a** ) Multiple giant gastric bezoars. ( **b and c** ) Mechanical lithotripsy was performed to fragment the bezoars, using a bezoar-specific lithotripter. ( **d** ) All the gastric bezoars were removed.

**Fig. 4:**
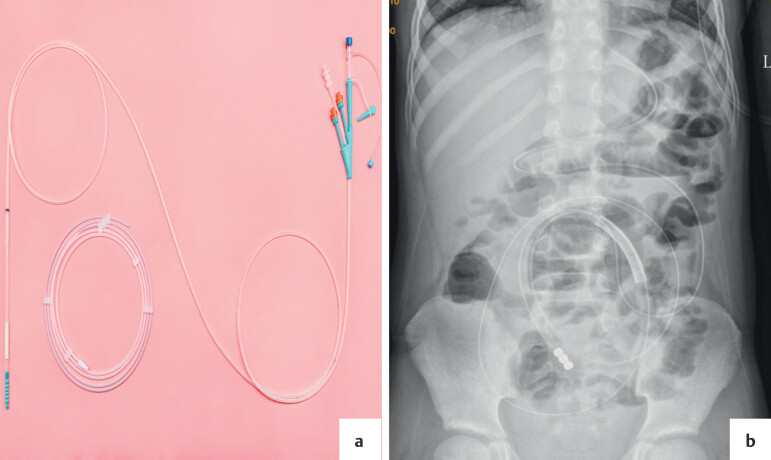
( **a** ) The schematic diagram of the ileus tube suite. The tube is 300 cm in length and 16 Fr. ( **b** ) The tube is advanced to the site of obstruction, intermittently aspirating the accumulated fluid and gas above the obstruction.

A 9-year-old boy presented with abdominal pain and vomiting for 5 days after eating hawthorn snacks on an empty stomach. Physical examination revealed upper abdominal pain, distension, and decreased bowel sounds. A computed tomographic scan showed multiple gastric calculi (largest 5.7×4.5×6.0 cm), extensive intestinal dilation, and a distal ileal stone (3.7×2.5×2.6 cm; Fig. 1 ). Initial conservative treatment with nasogastric Cola beverage infusion (500–1,000 mL/d for 2 d) softened the gastric stones. Subsequent gastroscopy was performed using a gastric stone cutter and crusher (WF-2417DTH, Fig. 2 ) to fragment the large bezoar into pieces of<2 cm, which were completely removed ( Fig. 3 ). To address the concurrent small bowel obstruction, a long ileus tube (16GB 3000TO, Fig. 4a ) was endoscopically placed in the jejunum and advanced to the obstruction site using its weighted tip ( Video 1 ). Intermittent aspiration reduced intestinal pressure and restored blood circulation . Cola beverage, sodium bicarbonate, and paraffin oil were infused through the tube. After 10 hours, the enterolith was successfully cleared, and abdominal distension resolved ( Fig. 4b ). The child recovered the normal bowel function and was discharged on a regular diet.

**Video 1** A case of combined endoscopic and long intestinal tube treatment for giant gastric bezoars with the ileus in a 9-year-old child.


Surgical intervention is typically necessary for gastric stones in the small bowel larger than 3 cm. Some suggest using a long ileus tube to improve conservative treatment success; 1​ however, its use in pediatric cases is poorly documented. This case illustrates that, for pediatric giant gastric bezoars complicated by ileus, a stepwise approach—initial endoscopic fragmentation and clearance followed by long tube decompression and dissolution—can achieve complete resolution. It highlights an effective endoscopic strategy that may obviate the need for surgery in selected pediatric patients.

Endoscopy_UCTN_Code_CCL_1AB_2AD_3AF
